# Caesarean Section Frequency among Immigrants, Second- and Third-Generation Women, and Non-Immigrants: Prospective Study in Berlin/Germany

**DOI:** 10.1371/journal.pone.0127489

**Published:** 2015-05-18

**Authors:** Matthias David, Theda Borde, Silke Brenne, Wolfgang Henrich, Jürgen Breckenkamp, Oliver Razum

**Affiliations:** 1 Charité University Medicine Berlin, Clinic for Gynaecology, Campus Virchow-Klinikum, Berlin, Germany; 2 Alice Salomon Hochschule Berlin—University of Applied Sciences, Berlin, Germany; 3 Charité University Medicine Berlin, Obstetrics Clinics, Campus Virchow-Klinikum and Mitte, Berlin, Germany; 4 Bielefeld University, School of Public Health, Department of Epidemiology & International Public Health, Bielefeld, Germany; Kuopio University Hospital, FINLAND

## Abstract

**Objective:**

The frequency of caesarean section delivery varies between countries and social groups. Among other factors, it is determined by the quality of obstetrics care. Rates of elective (planned) and emergency (in-labor) caesareans may also vary between immigrants (first generation), their offspring (second- and third-generation women), and non-immigrants because of access and language barriers. Other important points to be considered are whether caesarean section indications and the neonatal outcomes differ in babies delivered by caesarean between immigrants, their offspring, and non-immigrants.

**Methods:**

A standardized interview on admission to delivery wards at three Berlin obstetric hospitals was performed in a 12-month period in 2011/2012. Questions on socio-demographic and care aspects and on migration (immigrated herself vs. second- and third-generation women vs. non-immigrant) and acculturation status were included. Data was linked with information from the expectant mothers’ antenatal records and with perinatal data routinely documented in the hospital. Regression modeling was used to adjust for age, parity and socio-economic status.

**Results:**

The caesarean section rates for immigrants, second- and third-generation women, and non-immigrant women were similar. Neither indications for caesarean section delivery nor neonatal outcomes showed statistically significant differences. The only difference found was a somewhat higher rate of crash caesarean sections per 100 births among first generation immigrants compared to non-immigrants.

**Conclusion:**

Unlike earlier German studies and current studies from other European countries, this study did not find an increased rate of caesarean sections among immigrants, as well as second- and third-generation women, with the possible exception of a small high-risk group. This indicates an equally high quality of perinatal care for women with and without a migration history.

## Introduction

From an obstetrics perspective, the past two decades have been marked by a constant increase in the proportion of deliveries by caesarean section in practically all industrialized European countries. Germany currently ranks 5th in Europe with 31% caesarean deliveries, after Italy, Portugal, Romania and Cyprus [1]. A caesarean section can be a life-saving intervention for mother and baby, but it continues to be associated with risks and should therefore be performed only in the event of maternal or fetal indications [2,3]. The rate of caesareans is regarded as an important indicator of obstetrics quality [4,5]. Based on data from countries with very low maternal and neonatal mortality, WHO recommended a caesarean section rate of 15% as optimal [6]. Higher rates are conceivable if, for example, there is an increased demand for caesareans among pregnant women; lower rates could be interpreted as a sign of under-provision of obstetric care. Literature analyses show that in addition to medical-obstetric and structural care factors, general social conditions and attitudes of the pregnant women/parents influence the caesarean rates [7].

A number of papers have reported differences between immigrants, their offspring, and the non-immigrant population of the receiving county in regard to important perinatal care parameters such as caesarean rates. Stress caused by migration, disintegration of existing social networks due to migration or with increasing acculturation, low social status, poor access to the health care system, language barriers and discrimination in the care system can be reasons for differences in caesarean rates and perinatal outcomes among immigrants [8,9].

In Germany, 15 million of the approximately 80.2 million inhabitants have a migration background (they either immigrated themselves or are offspring of immigrants). That corresponds to almost 19% of the total population. In Berlin, the largest city, 23.9% of the residential population have a migration background [10]. The first papers on the subject of "births among foreigners" were published in Germany in the mid 1960s. They found a higher rate of surgical vaginal and caesarean deliveries and assumed that language barriers and communication difficulties had a negative influence on the childbirth process [11]. In the past 20 years only few large prospective studies have been conducted in Germany and in Europe which investigated whether factors associated with migration lead to a higher rate of surgical deliveries, in particular caesarean sections.

The decision to perform a caesarean delivery is complex. It depends on the birth situation and the assessment of clinical risks for the patient. At least for an elective (planned) caesarean the views and wishes of the patients influence the choice of the mode of delivery [12]. Socio-cultural influences from the country of origin and the degree of acculturation could also play a role here. From a clinical perspective, after adjustment for socio-demographic factors, the overall caesarean rates should not differ between immigrants and non-immigrants. One aim of the study was to examine whether and how migration status (immigrant, second- or third-generation, non-immigrant) affects the risk of an elective and of an emergency (in-labor) caesarean section, respectively. We examined intrapartum, neonatal and postpartum processes and outcomes along the following six questions: (1) Are the rates of elective and emergency caesarean sections different after adjustment for relevant clinical and socio-demographic factors? (2) Are there differences depending on the country or region of origin of the pregnant women? (3) What is the proportion of crash caesareans among emergency caesareans in the respective groups? (4) Do the factors acculturation, German language proficiency and education influence the frequency of the planned caesareans? (5) Are there differences in caesarean indications? (6) Do perinatal results differ in newborns delivered by caesarean, depending on the migration status of their mother?

## Methodology

Data were collected in three public Berlin obstetric hospitals (Charité Campus Virchow-Klinikum, Vivantes Klinikum am Urban, Vivantes Klinikum Neukölln). It was possible to carry out standardized interviews based on validated questionnaire sets translated into several languages with almost all women admitted to the three hospitals at least a few hours prior to the onset of labor pain. These primary data were later linked to the women’s antenatal records and to the perinatal obstetric data documented in the maternity wards for every birth. All hospitals throughout Germany have to regularly report these perinatal data to AQUA-Institut GmbH in Göttingen for quality assurance purposes. These data, however, do not contain sufficient information on socio-demographics and migration status.

The three-part study questionnaire set comprised 23 questions on socio-demographic aspects, 9 questions on care aspects and 23 questions on migration and acculturation based on a validated instrument (Frankfurt Acculturation Scale, FRAKK [13]). German language proficiency was self-assessed (“high” if German was first language or German skills were rated at least adequate; else “low”); net household income was self-reported by the woman (see Table 1 for categories).

**Table 1:** Socio-demographic factors by migration status.

	Immigrants 1st generation	2nd + 3rd generation women	Non-immigrants	All women
Total	2821 (39.7%)	958 (13.5%)	3321 (46.8%)	7100
**Age groups**				
18–24	592 (21.0%)	321 (33.5%)	561 (16.9%)	1474 (20.8%)
25–29	837 (29.7%)	306 (31.9%)	815 (24.5%)	1958 (27.6%)
30–34	770 (27.3%)	210 (21.9%)	1075 (32.4%)	2055 (28.9%)
35–49	622 (22.1%)	121 (12.6%)	870 (26.2%)	1613 (22.7%)
*Average*	*29*.*8*	*27*.*6*	*30*.*8*	*30*.*0*
missing values				0
**German language proficiency (self-assessed)**				
high	2017 (73.0%)	898 (98.9%)		
low	745 (37.0%)	10 (1.1%)		
missing values				109
**Level of education**				
Low*	684 (24.8%)	89 (9.3%)	111 (3.3%)	884 (12.6%)
Intermediate**	1119 (40.6%)	705 (73.9%)	1558 (47.1%)	3382 (48.2%)
High***	952 (34.6%)	160 (16.8%)	1641 (49.6%)	2753 (39.2%)
missing values				81
**Net household income**				
<€900	628 (26.4%)	197 (22.3%)	367 (11.8%)	1192 (18.7%)
€900 - €1500	982 (41.4%)	369 (41.7%)	706 (22.6%)	2057 (32.3%)
>€1500 - €2600	490 (20.7%)	217 (24.5%)	885 (28.4%)	1592 (25.0%)
>€2600	273 (11.5%)	102 (11.5%)	1160 (37.2%)	1535 (24.1%)
missing values				724
Acculturation****				
low	944 (34.3%)	153 (16.0%)	-	1094 (29.6%)
average	1315 (47.7%)	513 (53.8%)	-	1828 (49.3%)
high	496 (18.0%)	288 (30.2%)	-	784 (21.1%)
missing values				70

* No qualifications/primary school

** Secondary/technical school/vocational school

*** A-level/vocational diploma/technical college/university

**** Measured using the Frankfurt Acculturation Scale FRAKK [13], only for immigrant women and women of 2^nd^ + 3^rd^ generation

Her migration status was determined based on the recommendations by Schenk et al. (2006) [14]: information on the parents' country of birth, length of time in Germany and native language. Women with migration background were classified as follows: 1^st^ generation or immigrant (migrated themselves), 2^nd^/3^rd^ generation (offspring of immigrants), and women with a one-sided migration background (one parent without and one parent with own migration experience; in the analyses, these women were assigned to the non-immigrants due to small numbers and similarities in findings). Women not falling under one of these categories were classified as non-immigrant. The acculturation score was categorized as “low” (≤25^th^ centile), “average” (>25^th^ to <75^th^ centile), and “high” (≥75^th^ centile). A pregnancy was scored as “high-risk” based on doctors’ assessment during antenatal care (standardized scoring list in the antenatal card, comprising e.g. previous miscarriage, pre-existing severe disease, diabetes, age below 18 years or above 35 years).

Beginning in January 2011, trained project workers conducted interviews every day for one year in the delivery rooms and maternity wards of the three hospitals. They enrolled all women admitted for delivery within the study period with a viable pregnancy from 24 weeks of gestation, provided the woman was at least 18 years old at the time of birth of her baby and permanently resident in Germany. Minors, tourists, non-resident, women terminating a pregnancy, and women with miscarriages or stillbirths (infant death ascertained at hospital admission and before the onset of labor) were excluded.

A multiple imputation procedure using polytomous (multinomial) regression analyses which is provided in the “IVEware” software package [15] was used to impute missing data on acculturation (in 16.2% of cases). Imputation was based on age group, migration status (immigrants vs. 2^nd^/3^rd^ generation), and level of education. The remaining 70 missing data on acculturation are due to missing items on educational level. Univariate, bivariate and multivariate analyses for evaluating the effects of migration and acculturation processes on pregnancy and birth were carried out with the statistics software SAS 9.2. The significance level was set at p<0.05. Linear regression models were used to check for multicollinearity of variables selected for logistic regression analyses (data not shown as no evidence for multicollinearity was found). Logistic regression models were extended by statistically significant interaction terms using the forward selection technique. Marginal probability differences were calculated which reflect the effect of one unit change in the independent variable on the probability that the dependent variable is one [16]. For example, in Table 2 the marginal probability difference of -0.003 for 1^st^ generation immigrants means that their probability of an emergency caesarean is about 0.3 percent points lower than for non-immigrants. Odds ratios and marginal probability differences for interaction terms are interpreted relative to the respective reference of the interaction term (Tables 2 and 3). For example, the first interaction term in Table 2 shows that the chance of an emergency caesarean section in primiparous women with a high-risk pregnancy is higher than for nulliparous women without high-risk pregnancy. For the number of emergency caesareans per 100 births, 95% confidence intervals for differences between binomial proportions (Wald statistics) were calculated (Table 4). The p-values shown in Table 5 were adjusted for multiple significance testing using the Bonferroni method.

**Table 2:** Odds of an emergency caesarean section, adjusted for socio-demographic and clinical characteristics (N = 6875).

Emergency caesarean	Yes = 1455					
	No = 5420					
**Main effects**		N	Odds ratio	95% confidence interval	p-value	Marginal probability difference
Non-immigrants		3293	1.00			
1st generation immigrants		2682	0.98	0.85–1.14	0.8264	-0.003
2nd + 3rd generation women		900	0.97	0.80–1.19	0.7866	-0.004
Age: 18–24		1424	1.00			
Age: 25–29		1881	1.22	1.01–1.48	0.0360	0.031
Age: 30–34		1999	1.52	1.25–1.85	<0.0001	0.066
Age: 35–49		1571	1.87	1.52–2.30	<0.0001	0.103
High level of education		2713	1.00			
Intermediate level of education		3307	1.04	0.90–1.20	0.5702	0.006
Low level of education		855	0.97	0.75–1.24	0.7846	-0.005
High German language proficiency		6163	1.00			
Low German language proficiency		712	0.87	0.68–1.11	0.2576	-0.021
High-risk pregnancy no		4309	1.00			
High-risk pregnancy yes		2566	1.19	1.01–1.41	0.0401	0.027
Nullipara (P0)		3214	1.00			
Primipara (P1)		2085	0.40	0.32–0.49	<0.0001	-0.128
Bipara (P2)		941	0.26	0.19–0.36	<0.0001	-0.154
Multipara (P3 or higher)		635	0.35	0.24–0.51	<0.0001	-0.126
Birth weight <2500 g		645	2.91	2.32–3.65	<0.0001	0.201
Birth weight 2500 - <4500 g		6149	1.00			
Birth weight > = 4500 g		81	7.15	2.93–17.48	0.0006	0.398
**Interaction terms**						
High-risk pregnancy no * nullipara		2125	1.00			
High-risk pregnancy yes * primipara		810	1.49	1.11–2.00	0.0080	0.064
High-risk pregnancy yes * bipara		382	1.50	0.97–2.31	0.0673	0.067
High-risk pregnancy yes * multipara		285	1.03	0.63–1.69	0.8962	0.005
Nullipara * birth weight 2500 - <4500 g		2824	1.00			
Primipara * birth weight <2500 g		143	1.76	1.15–2.69	0.0089	0.097
Primipara * birth weight > = 4500 g		29	0.13	0.03–0.54	0.0048	-0.173
Bipara * birth weight <2500 g		81	4.32	2.48–7.52	<0.0001	0.289
Bipara * birth weight > = 4500 g		14	0.53	0.12–2.38	0.4095	-0.080
Multipara * birth weight <2500 g		55	3.07	1.62–5.83	0.0006	0.212
Multipara * birth weight > = 4500 g		14	0.16	0.03–0.92	0.0398	-0.167

Marginal probability difference reflects the effect of one unit change in the independent variable (e.g. non-immigrants = 0, 1^st^ generation migrants = 1) on the probability that the dependent variable is one (= emergency caesarean) [16]

**Table 3:** Odds of an elective caesarean section, adjusted for socio-demographic and clinical characteristics (N = 6875).

Elective caesarean	Yes = 918					
	No = 5957					
**Main effects**		N	Odds ratio	95% confidence interval	p-value	Marginal probability difference
Non-immigrants		3293	1.00			
1st generation immigrants		2682	0.88	0.74–1.05	0.1551	-0.014
2nd + 3rd generation women		900	0.80	0.63–1.02	0.0684	-0.023
Age: 18–24		1424	1.00			
Age: 25–29		1881	1.71	1.12–2.59	0.0127	0.030
Age: 30–34		1999	2.09	1.32–2.18	0.0006	0.040
Age: 35–49		1571	4.01	2.61–6.17	<0.0001	0.075
High level of education		2713	1.00			
Intermediate level of education		3307	1.07	0.91–1.27	0.3933	0.008
Low level of education		855	0.73	0.53–0.98	0.0390	-0.033
High-risk pregnancy no		4309	1.00			
High-risk pregnancy yes		2566	3.09	2.10–4.53	<0.0001	0.135
Nullipara (P0)		3214	1.00			
Primipara (P1)		2085	2.18	1.42–3.34	0.0003	0.095
Bipara (P2)		941	2.35	1.27–4.33	0.0063	0.115
Multipara (P3 or higher)		635	0.48	0.06–3.72	0.4802	-0.067
High German language proficiency		6163	1.00			
Low German language proficiency		712	0.92	0.69–1.23	0.5778	-0.009
Birth weight <2500 g		645	1.88	1.41–2.51	<0.0001	0.082
Birth weight 2500 - <4500 g		6149	1.00			
Birth weight > = 4500 g		81	0.29	0.04–2.20	0.2317	-0.090
**Interaction terms**						
High-risk pregnancy no * age: 18–24		1013	1.00			
High-risk pregnancy yes * age: 25–29		633	0.74	0.46–1.19	0.2166	-0.031
High-risk pregnancy yes * age: 30–34		730	0.70	0.44–1.10	0.1224	-0.037
High-risk pregnancy yes * age: 35–49		792	0.51	0.32–0.81	0.0042	0.064
Age: 18–24 * nullipara		914	1.00			
Age: 25–29 * primipara		567	0.86	0.51–1.46	0.5863	-0.016
Age: 25–29 * bipara		262	0.57	0.27–1.20	0.1408	-0.052
Age: 25–29 * multipara		139	2.21	0.26–18.58	0.4666	0.111
Age: 30–34 * primipara		668	0.87	0.52–1.44	0.5788	-0.015
Age: 30–34 * bipara		297	0.74	0.36–1.50	0.4022	-0.031
Age: 30–34 * multipara		214	1.59	0.19–13.08	0.6672	0.059
Age: 30–49 * primipara		495	0.45	0.27–0.77	0.0031	-0.071
Age: 30–49 * bipara		259	0.51	0.25–1.05	0.0663	-0.060
Age: 30–49 * multipara		214	1.11	0.14–8.96	0.9233	0.012
Nullipara * birth weight 2500 - <4500 g		2824	1.00			
Primipara * birth weight <2500 g		143	0,52	0,31–0.89	0.0165	-0.058
Primipara * birth weight > = 4500 g		29	2.60	0.26–25.74	0.4146	0.141
Bipara * birth weight <2500 g		81	0.60	0.31–1.16	0.1294	-0.047
Multipara * birth weight <2500 g		55	0.77	0.31–1.92	0.5798	-0.026
Bi-/Multipara * birth weight > = 4500 g		28	4.47	0.41–48.20	0.2172	0.358

Marginal probability difference reflects the effect of one unit change in the independent variable (e.g. non-immigrants = 0, 1^st^ generation migrants = 1) on the probability that the dependent variable is one (= elective caesarean) [16]

**Table 4:** Number of crash and emergency caesareans, and proportion of crash caesareans per 100 births, by migration status.

	Immigrants 1st generation	Women 2nd + 3rd gen.	Non-immigrants	All women

Crash caesarean sections	52	10	50	112
	(10.1%)	(5.6%)	(6.3%)	(7.5%)
Emergency caesarean sections	461	170	747	1378
(excl. crash caesareans)	(89.9%)	(94.4%)	(93.7%)	(92.5%)
No. of crash caesareans per	1.84	1.04	1.51	1.58
100births (95% confidence interval)	(1.35–2.34)	(0.40–1.69)	(1.09–1.99)	

**Table 5:** Perinatal outcomes by mode of delivery and by migration status.

	Immigrants		Non-immigrants	All
	1st generation	2nd + 3rd gen.		women
**Arterial umbilical cord pH-value >7.10**				
Vaginal birth	1925 (98.2%) p = 0.0066	646 (96.6%) p = 1.0000	1947 (96.7%) Reference	4518 (97.3%)
Elective caesarean	332 (98.2%) p = 1.0000	107 (100.0%) p = 0.4463	494 (98.0%) Reference	933 (98.3%)
Emergency caesarean	489 (96.3%) p = 1.0000	178 (99.4%) p = 0.0889	768 (96.6%) Reference	1435 (96.8%)
5 min Apgar score > = 7 *					
Vaginal birth	1956 (99.3%) p = 0.2637	666 (99.3%) p = 1.0000	1996 (98.9%) Reference	4618 (99.1%)
Elective caesarean	333 (98.2%) p = 1.0000	101 (94.4%) p = 0.2120	494 (97.6%) Reference	927 (97.5%)
Emergency caesarean	483 (94.2%) p = 1.0000	173 (96.1%) p = 0.7340	750 (94.1%) Reference	1406 (94.4%)
**Transfer to pediatric clinic**					
Vaginal birth	176 (8.9%) p = 0.0038	63 (9.4%) p = 0.1357	242 (11.4%) Reference	481 (10.3%)
Elective caesarean	71 (20.9%) p = 0.1007	28 (26.2%) p = 1.0000	136 (26.9%) Reference	235 (24.7%)
Emergency caesarean	176 (34.3%) p = 0.5025	42 (23.3%) p = 0.0760	249 (32.2%) Reference	467 (31.4%)

Notes:

* Study possibly underpowered for this outcome (see Discussion section).

Adjusted for multiple significance tests using the Bonferroni method. P-value in bold: statistically significant p<0.05

Ethical clearance was obtained from the Berlin Charité Ethics Committee (Ethikkommission I, Campus Charité Mitte, dated 18 Feb 2009). All participants provided their written consent on a form cleared by the Charité Ethics Committee. Data protection regulations were also cleared and observed.

## Results

In the study period 8157 women gave birth in the three Berlin obstetrics hospitals. It was not possible to contact 363 women despite multiple attempts. Of the 7794 women invited to participate, only 381 (4.9%) declined to be interviewed; 235 women (3.0%) met one of the exclusion criteria. Six women (< 0.1%) did not consent to linking the interview data with the clinical/obstetric data. In 72 cases (1.0%) it was not possible to merge the clinical perinatal data with the interview data. Ultimately, data from a total of 7100 women were available for analysis, corresponding to a response rate of 89.6%. The questionnaire data of the 7100 women related to 7334 birth data records due to twin and triplet births. For the analysis, the mother's data and the data of her newborn or of her first-born in case of a multiple birth were considered. Among the women interviewed, 39.7% were immigrants and 13.5% belonged to the second and third generation. On account of their small number (n = 11), 3^rd^ generation women were grouped together with 2nd generation women in the analysis. Table 1 shows the main socio-demographic data of the three groups studied.

Table 6 details the frequency of vaginal (spontaneous and surgical) and caesarean deliveries (elective and emergency) in the three groups. Only small differences are visible.

**Table 6:** Modes of delivery by migration status (numbers and proportion in %).

	Immigrants 1st generation	2nd + 3^rd^ generation women	Non-immigrants	All women
	
Vaginal birth*	1969 (69.8%)	671 (70.0%)	2019 (60.8%)	4659 (65.6%)
Elective caesarean	339 (12.0%)	107 (11.2%)	505 (15.2%)	951 (13.4%)
Emergency caesarean	513 (18.2%)	180 (18.8%)	797 (24.0%)	1490 (21.0%)
Total	2821 (100%)	958 (100%)	3321 (100%)	7100 (100%)

The results of logistic regression analyses in Tables 2 and 3 show that even after adjustment for clinical and socio-demographic factors there are no significant differences between the three groups with regard to the risk of an elective / emergency caesarean delivery (N = 6875 pregnant women with full specification of all parameters). The tables show that the age of the pregnant woman, a high-risk pregnancy, parity, and the birth weight of the baby have a significant effect on the odds of caesarean section while the migration status of the woman does not.

The analysis of interaction shows that primiparous and multiparous women with low birth weight infants have higher odds of an emergency caesarean section compared to the reference group (nullipara * birth weight 2500-<4500g; see Table 3). Women with high birth weight infants have lower odds of an emergency caesarean section. For elective caesarean sections, the opposite is the case. The interaction age * parity was significant only for elective caesarean section, see Table 3. This indicates that younger nulliparous women as well as older multiparous women have the highest odds of undergoing an elective caesarean section.

Table 7 shows the mode of delivery for first generation immigrants (n = 2802) by country / region of origin, stratified by mode of delivery. There are clear (and in many cases statistically significant) differences, especially with regard to emergency caesareans which are more frequent among women from sub-Saharan Africa and from Latin America.

**Table 7:** Mode of delivery for 1^st^ generation immigrants by region/country of origin (n = 2802, 19 missings).

	N	Vaginal birth (%)	Elective caesarean (%)	Emergency caesarean (%)
Turkey	697	78.1	10.5	11.5
"Europe 15" and EFTA	139 ***	61.9	7.2	30.9
Other Europe (excluding Yugoslavia)	446 ***	67.5	13.7	18.8
Former Yugoslavia and Albania	309 *	72.2	9.7	18.1
Arab countries and Israel	151 *	68.9	15.9	15.2
Lebanon	378 ^n.s.^	77.8	10.3	11.9
North, Central, and South Africa	213 ***	51.7	15.0	33.3
CIS (neighbor) countries	238 **	67.7	13.5	18.9
India and other Asia	124 ***	62.9	16.1	21.0
Other countries	77 ***	52.0	15.6	32.5
All	2802 ^n.s.^	69.8	12.0	18.1

Chi^2^ test for heterogeneity:

* p<0.05,

** p<0.01,

*** p<0.001 (reference: Turkish immigrants)

Row percentages different from 100% are due to rounding.

Details on the country groups:

(1) "Europe 15": Belgium, Luxembourg, Holland, Germany, France, Italy, Denmark incl. Greenland, United Kingdom, Ireland, Greece, Spain (incl. Canaries, Ceuta, Melilla), Portugal, Sweden, Finland, Austria

(2) EFTA (European Free Trade Association): Iceland, Norway, Switzerland, Liechtenstein

(3) Other Europe: Poland, Romania, Bulgaria, Slovakia, Slovenia, Czech Republic, Hungary, Latvia, Lithuania, Estonia, Malta, Cyprus & Cypriot Republic,

(4) Former Yugoslavia: Croatia, Serbia, Bosnia-Herzegovina, Montenegro, Macedonia, Albania,

(5) CIS (neighbor) countries: Russia, Belarus, Ukraine, Moldavia, Azerbaijan, Armenia, Kazakhstan, Kyrgyzstan, Tajikistan, Turkmenistan, Uzbekistan, Georgia, Afghanistan, Pakistan

(6) Arab countries: Saudi Arabia, Yemen, Oman, United Arab Emirates, Bahrain, Qatar, Iraq, Iran, Jordan, Syria

(7) North Africa, Central and South Africa: Morocco & West Sahara, Algeria, Tunisia, Libya, Egypt, Mauretania, Mali, Niger, Chad, Sudan, South Africa

(8) India and the rest of Asia: India, Nepal, Bangladesh, Bhutan, Myanmar, Thailand, Cambodia, Vietnam, Malaysia, Indonesia, Brunei, Philippines, Papua New Guinea, Salomon Islands, Laos, North Korea, South Korea, Taiwan, Japan

(9) Other countries: China and Mongolia, Oceania, USA, Canada and mini-states, Latin America and Caribbean

The most frequent indication for an elective caesarean in all three groups examined was”previous caesarean or other uterus operation“, followed by the indication”breech presentation“. In all three groups, emergency caesarean deliveries were performed most frequently due to”pathological cardiotocography (CTG)”.”Condition after caesarean”and”protracted birth/obstructed labor in the opening period”followed second and third on the indication list in all three groups (these findings are not shown in the tables).

The influence of the factors acculturation, German language proficiency and education on the risk of an elective caesarean was compared between the immigrant and the 2^nd^/3^rd^ generation groups (data not shown in the tables). The only noteworthy difference (albeit not statistically significant) was found with regard to school-leaving qualifications: women without any school-leaving qualifications or with primary education only seem less likely to undergo an elective caesarean than those with intermediate or higher school-leaving qualifications (OR 0.78; 95% CI 0.54–1.11).

Table 4 shows the proportion of crash caesareans among emergency caesareans in the three groups. Logistic regression modeling of the odds of women undergoing a crash caesarean did not indicate any statistically significant differences between the three groups (data not shown). The total crash caesarean rate was 1.58 per 100 births, with slightly higher rates among first generation immigrants (18.4) than among non-immigrants (15.1). The differing rate in the sub-group with < = 10 emergency caesareans is probably a random effect due to the small number of cases.

In Table 5 the neonatal outcomes are summarized, stratified by mode of delivery (vaginal birth, elective and emergency caesarean) and migration status. The frequency of adverse arterial umbilical cord pH-values <7.10 differs minimally between the three groups studied. There was only a significant difference in the rate of transfer of the newborn to a pediatric clinic or neonatal department after vaginal delivery in 1^st^ generation immigrants relative to the non-immigrant group (reference; p = 0.0038).

## Discussion

### Principal findings

The caesarean section rate is regarded as an important indicator for assessing process quality of obstetrics care [4,17]. At the same time, ongoing discussions about "requested caesareans" show that various medical and non-medical factors at least partly influence the rate of caesarean sections [18,19]). The results of our study show that the caesarean rates of the immigrant women, of the 2^nd^/3^rd^ generation women, and of the non-immigrant women in a large sample of consecutive births in Berlin/Germany were similar (albeit at a high level), with similar indications leading to caesarean deliveries and with near-equal neonatal outcomes. While this study did not find an overall increased rate of caesareans among immigrants and their offspring, the rate of crash caesareans per 100 births was somewhat higher among 1^st^ generation immigrants than among non-immigrants. This is probably due to barriers to access to care experienced by a small sub-group of presumably less acculturated pregnant women.

### Strengths and limitations

The strengths of the study are the prospective data collection, the availability of detailed data e. g on antenatal care and acculturation, and the high participation rate. For the first time in an epidemiological perinatal study carried out in Germany a questionnaire to gather precise information on migration status and acculturation was used, and for the first time were the issues of caesarean frequency, migration and birth outcome analyzed together. In this way, the clinical perinatal data could be supplemented by important socio-demographic data. A methodological limitation of the study is its sample size (which is small when compared to national register studies such as Vangen et al. 2000 [20]; Sørbye et al. 2014 [21]). A before-hand power analysis showed that power was sufficient (>80–90%) to demonstrate relevant differences in the main maternal outcomes; a post-hoc power analysis showed that it was also sufficient for two of the neonatal outcomes (pH and transfer; 85% and 75%) but not for the (very small) difference in Apgar score. Another concern is the documentation quality of the perinatal data collected routinely in the obstetrics clinics. Furthermore, there was substantial heterogeneity regarding region of origin in the sample (see Table 7), which may have attenuated differences between sub-groups. Finally, the results from a city with a high proportion of immigrants cannot be generalized to smaller towns or rural areas with a low proportion of immigrants in the population. But despite methodological criticism it can be said that our results currently provide the most valid picture of caesarean section frequency, indication and outcomes among immigrants in Germany.

### Comparison with other studies

The postnatal condition of newborns has rarely been a focus of other publications on immigrants or their descendants; rather, the differences were almost always discussed among a (specialist) audience regarding the frequency of caesareans and their interpretation (for example, see Merry et al. 2013 [22]). This means that few comparative data are available regarding the birth outcome. Moreover, the high overall caesarean section rate in our sample needs to be taken into account when making comparisons to other countries, which may have lower overall rates.

Margioula-Siarkou et al. (2013) [23] published a retrospective analysis of approximately 7000 births in a Greek clinic. 47.6% of the women were immigrants, the rate of emergency caesareans among them was somewhat lower and babies born to the immigrant group had better overall Apgar scores 1 and 5 min. postpartum. In 2004 in Italy Rizzo et al. [24] established more frequent "planned caesareans" as a mode of delivery among native women compared with immigrants living there. Our study, however, did not find this difference between immigrants and non-immigrants. An analysis of birth data of approximately 1800 newborns collected prospectively in Austria showed a significant difference only for one sub-group, namely women of Turkish origin, who had fewer elective caesarean deliveries and more vaginal births compared with non-immigrant women [25]. Several authors have established a higher frequency of emergency caesarean deliveries among immigrants and ethnic minorities in general [26–29]. However, the caesarean frequency often differs in sub-groups only, according e.g. to country of origin or duration of stay [21]. In their analysis of the Norwegian birth register Vangen et al. [20] found a higher rate of caesareans among immigrants from the Indian sub-continent, Africa and Latin America, but the frequency among other immigrants e.g. from Turkey or Pakistan was at a similar low level to native Norwegian women. Rio et al. (2010) [4] reported on an analysis of data that was also based on (Spanish) register data and were able to show that the risk for immigrants of a caesarean delivery is lower than for non-immigrant Spanish women. However, the risk was slightly higher for particular regions, e.g. for immigrants from Latin America.

Our analysis according to regions of origin also shows considerable differences, especially in the rate of emergency caesareans for first generation immigrants. Alongside women who immigrated from countries of the European Union, immigrants from Africa and Latin America [4,20] had the highest caesarean rates, in spite of much lower rates in the regions of origin (sub-Sahara Africa 4%, Latin America/Caribbean 23.7%) [5]. Reasons may include distinct attitudes to caesarean section as a mode of delivery, different maternal knowledge and views on the management of deliveries, and diverging routines of obstetric personnel [30,31]. The differences found in the studies originating from Europe [32,33] can possibly be explained by the heterogeneity of the immigrant populations in the various European countries, differences in health reporting and different data collection and recording (definition of target group by country of birth, migration background, or ethnicity).

Oberaigner et al. (2013) [25] examined whether factors associated with migration affect the frequency of caesareans. Unlike in our analysis, Oberaigner et al. identified language proficiency as a relevant parameter: the proportion of vaginal births was significantly higher in the sub-groups of women from ex-Yugoslavia and Turkey. It decreased with increasing length of residence in Austria, and with increasing German language proficiency; the proportion of elective caesareans was highest in the non-immigrant group. Here, too, there were differences between the immigrant groups and a significant increase in the rate of elective caesareans with increasing length of residence in Austria and German language proficiency. The proportion of emergency caesareans was largely the same for all immigrant groups and independent of the length of residence in the country [25].

### Implications

Even though there has been a nationwide, obligatory quality assurance system for all obstetrics clinics in Germany for more than twenty years, to the present day no information on the country of origin of the pregnant women or other important socio-demographic parameters are recorded that would enable further scientific analyses, for example of the influence of migration and acculturation processes on pregnancy and birth [34]. With the study presented here, for the first time such data was collected prospectively and representatively for an urban centre of Germany. Unlike in register studies from Scandinavian countries, it was also possible to consider the degree of acculturation. In our study group, the migration status is not associated with a significantly higher risk of a caesarean delivery. Together with the similar neonatal outcomes after caesarean deliveries (umbilical cord pH and Apgar score, transfer rate of newborns), this indicates equally good standards of medical care at Berlin obstetrics clinics for immigrants, second- and third-generation women as well as non-immigrants. This may apply also to other German cities and industrial regions. However, in view of the international literature, differences in care cannot be ruled out in smaller towns and especially in rural areas with a low proportion of immigrants in the population. This should be examined prospectively in a multicentre study of several urban and rural hospitals.
